# 72 Years of Homemaking in Waiting Zones: Lebanon's “Permanently Temporary” Palestinian Refugee Camps

**DOI:** 10.3389/fsoc.2020.587063

**Published:** 2020-11-26

**Authors:** Yafa El Masri

**Affiliations:** Department of Historical and Geographic Sciences and the Ancient World, University of Padua, Padua, Italy

**Keywords:** Palestinian refugees, refugee camps, protracted refugees, livelihoods, homemaking, statelessness

## Abstract

The “permanently temporary” Palestinian refugee community, present in Lebanon since 1948 with no solution in sight, has the highest rate of abject poverty within all five areas of operation of United Nations Relief and Works Agency for Palestinians in the Near East (UNRWA), and it still occupies the same limited geographic space it did 72 years ago. This harsh reality stems from the refugees' statelessness but is also worsened by the local conditions imposed by the Lebanese legislation and (non)settlement policy aimed at preventing refugees from becoming permanent. Within this situation, we look at practices of agency enacted by camp dwellers to provide lacking life necessities and improve living conditions in the camps. This paper will identify and analyze coping mechanisms and homemaking practices undertaken by Palestinian refugees in Lebanon, operating within the framework set by Brun and Fábos (2015), which conceptualized home and homemaking for people in protracted displacement through identifying a refugee's “home, Home and HOME.” Building on Donna Haraway's concept of situated knowledge, this paper uses data collected from participant observations, interviews, ethnographic and autoethnographic recordings analyzed through the lens of my own positioned rationality as a Palestinian refugee from Lebanon. Further, the paper will explore how Palestinian refugees establish camp spaces as a “home-Home-HOME,” despite their uncertain futures, through vertical expansion of buildings, stories and family bonding, in addition to trading and micro-markets. The paper will also deduce how refugees' informal coping mechanisms offer a way of strengthening community bonds, making home in those, otherwise, uncomfortable “waiting zones,” and finally, envisioning new ideas for restructuring the camp beyond the rule of formal institutions.

*Najwa gazes at the green fields beyond the UNIFEL Blue Line that separates Lebanon from the occupied Palestinian territories. She points to the far lands, turns her head towards me and says, “My dignity is there.” She continues to narrate how her “life in Lebanon's refugee camps will always be incomplete,” and that they are only a waiting zone. For Najwa, the only permanence she can recognize exists in her Palestinian homeland, and her “life could only be completely dignified in the original home,” referring to the homeland that she has never visited*.

## Why, We Refugees, Write

“*The moment we arrived to Saida [City in the South of Lebanon] in the afternoon, we became refugees”*- (Kanafani, 2015, p. 75).

Even though I agree with Hannah Arendt that “We don't like to be called refugees”(Arendt, 1943, p. 264), I also strongly agree with her statement that the process of thought, let alone the process of research can seldom be possible without being attributed to a personal experience (Arendt, 1964). It is thus necessary to contextualize that the initial motive for this paper stems from my background as a Palestinian refugee.

I grew up and spent most of my life in Lebanon's Palestinian refugee camps between 1990 and 2016. I was born in a refugee camp in Lebanon to two Palestinian refugee parents and was automatically granted Palestinian refugee status, a big blue refugee document bearing the name of the city we come from in Palestine (Yafa in Arabic, Jaffa In English), but I was never given the citizenship of any nation.

My personal background is worth mentioning in this introduction because this project derives from my situated knowledge within the Borj Albaranejah refugee camp, which has inevitably influenced my view of the refugee crisis. Defined as the knowledge that reflects the perspectives of the knower, situated knowledge is significant for feminist epistemology as a whole. Donna Haraway views that all eyes, including our own organic ones, are active perceptual systems, building on translations and specific ways of seeing things, simply because there is no unmediated image of any object/phenomenon (Haraway, 1988). According to Haraway, one must situate the view of an image from the perspective of the subjugated in order to see well (Haraway, 1988) and that “by acknowledging and understanding the contingency of their own position in the world, and hence the contestable nature of their claims to knowledge, subjects can produce knowledge with greater objectivity than if they claimed to be neutral observers” (Haraway, 1988, p. 583). Haraway here legitimizes the use of personal positionality and situated passion. Rather, she rather argues the necessity of a doctrine and the practice of objectivity in science that privileges contestation, deconstruction, passionate construction, webbed connections, and she hopes for transformative systems of knowledge and ways of seeing, advocating for situated and embodied knowledges that are locatable and accountable. As such, coming from the lived experience of the subjugated, I align my own methodology with Haraway's feminist approach of viewing science and refugee studies through positioned rationality, based on situated knowledges.

Like my own family, thousands of Palestinians were forcefully displaced from their Palestinian villages and towns in 1948. Some Palestinians were internally displaced to Gaza and the West Bank, whereas others were externally displaced to neighboring countries. The displacement of Palestinians and the birth of the Palestinian refugee crisis can be best summarized by Walid Alkhalidi who claims that the only problem with establishing a homeland for the Jews in Palestine was that *the land was already inhabited* (Khalidi, 2006). Israeli historian Benny Morris has researched extensively for years and reported at length on the military attacks and brutal expulsions performed by Israeli groups that forced Palestinians out of their lands in 1948 (Morris, 2004). As a result of this conflict over the land, the Palestinian community went through what the Israeli Historian Illan Pappe names “The Ethnic Cleansing of Palestinians” (Pappe, 2015). In 1948, three quarters of a million Palestinians were forced to seek refuge in surrounding countries, such as Lebanon, Jordan, Syria or Egypt and became known as Palestinian Refugees: a community of an estimated 5 million people today (UNRWA, 2020a).

Despite lasting 72 years, the Palestinian-Israeli conflict and its consequences have been a hot topic for both political and social studies worldwide. More specifically, the Palestinian refugee problem has been studied for its complex humanitarian situation, in which Palestinian refugees are the world's only exception to the international protection regime (United Higher Commissioner for Refugees- UNHCR) and are therefore some of the most vulnerable displaced groups in the world (Shiblak, 2006). However, this paper aims to deviate from the mainstream view of Palestinian refugees (and refugees in general) as helpless victims, legitimate targets of international assistance, or solely as passive recipients of humanitarian services. My contribution sheds the light on the agency of Palestinian refugees in establishing their own home in a space of waiting and in devising non-conventional methods of survival while waiting. Such an approach not only explores the existence of Palestinian refugees as actors and creators but also takes on Haraway's perspective of situated knowledge, wherein she argues for necessity of the object of knowledge function to be both an actor and an agent (Haraway, 1988).

First, I will explain my understanding of the theoretical framework, which conceptualizes homemaking *in situ*ations of protracted refugees. I will then explore the underreported daily and long terms practices of Palestinian refugees in their spaces of dwelling. Finally, I will map these practices within the three categories of the aforementioned homemaking framework, followed by a conclusion and reflections on this final distribution of practices within the homemaking framework.

## From Spaces of Exception to Potential Homes: A Literature Review

After being removed from their homes, Palestinian refugees have become stateless persons in their new host communities. This process of denationalization (becoming stateless) is best explained by Hannah Arendt, who states that the removal of people from organized political communities leaves them with “no country on earth in which they enjoyed the right to residence” (Arendt, 1968, p. 276). These people are no longer citizens of a sovereign state (stateless non-persons as Arendt refers to them); “nobody wants to claim them, and therefore their lives may be in danger”(Arendt, 1968, p 240). She explains how the invalidity of man's rights leads to the “silent consents to unprecedented conditions” (Arendt, 1968, p. 447). This view leads us to understand the unique social realities in which refugees around the world continue to live despite contradicting the International humanitarian declarations and political obligations toward preserving human dignity. Similarly, stateless refugees could be represented through the views of Giorgio Agamben, who extensively describes the life of a Homo Sacer and the states of exception. *Homo sacer* is defined in legal terms as someone who can be killed without the killer being named a murderer, as well as a person who cannot be sacrificed (Agamben, 1998). This person is excluded from society and deprived of basic rights and all functions in civil religion (Agamben, 1998). Therefore, the Homo sacer may thus be understood as someone existing outside of the law, or beyond it, in a state of exception, which is precisely the stateless person, whose termination can happen through many ways (Agamben, 1998). With both views here overlapping, it becomes clear how a refugee's lack of protection allows the violation of basic human rights. Even though mechanisms are theoretically in place to safeguard those rights, lack of protection by a recognized state deprives the refugee from an actual implementation of such framework that empirically guarantees his dignity and rights.

In many cases, refugees remain stuck in a space of exception, being the Homo sacer, for long decades, thus becoming “Protracted Refugees.” UNHCR defines a protracted refugee as:

“One in which refugees find themselves in a long-lasting and intractable state of limbo. Their lives may not be at risk, but their basic rights and essential economic, social and psychological needs remain unfulfilled after years in exile. A refugee in this situation is often unable to break free from enforced reliance on external assistance.” (UNHCR, 2004, p. 1)

In exploring the lives of groups in limbo, Anita Fábos and Cathrine Brun affirm that host nations largely have an interest in keeping people in a temporary status for long periods of time. People who are forced to flee tend to occupy these marginal spaces [refugee camps] because they are considered a threat to the social and political order (Brun and Fábos, 2015). More importantly, the framework set by Fábos and Brun views temporary refugee camps as home to protracted refugees. The authors analyze home both as an idea (or even an emotional perspective) and a practice by outlining three different yet interrelated elements:

- “home” being the day-to-day practices of homemaking. These practices include improvements and investments that people make to their temporary dwellings in order to improve their quality of living. But they can also include the daily routines that become habitual for people and incorporate social connections with the surrounding.
- “Home” as the set of values, traditions, memories, and the emotional sense of belonging. This infers a subjective feeling of home, holding much nostalgia, regardless of the physical distance between the home-maker and his home. In other words, this notion of Home is constructed in regards to the place that the displaced group longs, dreams and thinks of when going about their lives and while constructing their temporary dwelling place.
- “HOME” is the broader political and historical contexts by which the camp dwellers stand in the current global order and embedded in institutions. It refers to the geopolitics of the homeland that have contributed or continue to contribute to the population's situation of protracted displacement. It is the interaction of the displaced group with the political arrangements of the homeland in a mutually influential manner.

To summarize, Brun and Fábos' framework of homemaking can be verified through the attempt to reconstruct a past familiarity, envision a better future and empirically improve one's living conditions in given place through a variety of practices. This framework allows us to analyze the process of homemaking in communities of protracted refugees by exploring the practices, views and even feelings of individuals in order to formulate an idea about their exceptional home-making efforts in the waiting zones. The authors also explain that refugees, through the simple yet unimaginably difficult tasks of living their lives, can contribute to the rethinking and production of durable solutions for their prolonged cases of displacement (Brun and Fábos, 2015). The empirical application of this idea will also be explored through observing how Palestinian refugees rethink and produce their own space of exile in the absence of permanent settlement plan and development assistance.

Similarly, Johannes Lenhard and Farhan Samanani, who aim to diversify the meanings of home by revealing articulations of home which take shape in unexpected places, view that the meaning of home changes depending on perspective. Home can be made of bricks and mortar, possessions, but it can also surpass the materiality of a dwelling place to represent feelings, stories or habits (Lenhard and Samanani, 2020). Through this concept, we may better explore how Palestinian refugees make home in their space of waiting not only observing material elements but also emotional and immaterial ones.

This contribution of refugees to shaping their own space of waiting recalls Michel Agier's views of refugee camps as waiting rooms, where the majority of refugees find themselves stuck in limbo for years; yet, it also confirms that refugee camps, established as spaces that freeze their inhabitants' status, transform their temporariness into a transient permanency (Agier, 2011). In other words, camps that are meant to stay fixed, evolve greatly, defying restriction and expanding in a manner such that refugees and inhabitants reproduce their own normality (Agier, 2011). A situation that we aim to explore is one that demostrates how Palestinian refugees attempt to produce a normality in which they can survive. Indeed, occupying a temporality between temporariness and permanency, Lebanon's refugee camps today can be said to inhabit ‘a “frozen transience,” or in the words of Bauman: an ongoing, long-lasting state of temporariness, a duration of periods just patched together by successive events (Bauman, 2002). But refugee camps do have the potential to partially develop their own scenarios and orchestrate certain extents of their reality. As Thrift (2006) states, space must be addressed as a process and not as a frozen materiality; it is never static but is always fluid and in constant motion (Thrift, 2006). Hence, when applying that to the refugee camp—a land understood as the permanent spatialization of the exception- we can see a constant dynamic development of the camp's symbolic and physical features. Such can be viewed not only in the replaced construction from fabric tents to concrete, but also in how people make a home in living within the camp space. The ways in which the camp can transform and develop are normally decided by the sovereign, but the circumstances and the people inhabiting the space or its surroundings, also decide with him, even if their contribution goes by unnoticed (Martin, 2015). These people here are primarily refugees and camp dwellers, and their unrecorded contribution to the reorganization of their space must be acknowledged and reinterpreted as an act of agency and homemaking.

## Context Overview: Presentation of the Case Study of Palestinian Refugees in Lebanon

**Figure 1:**
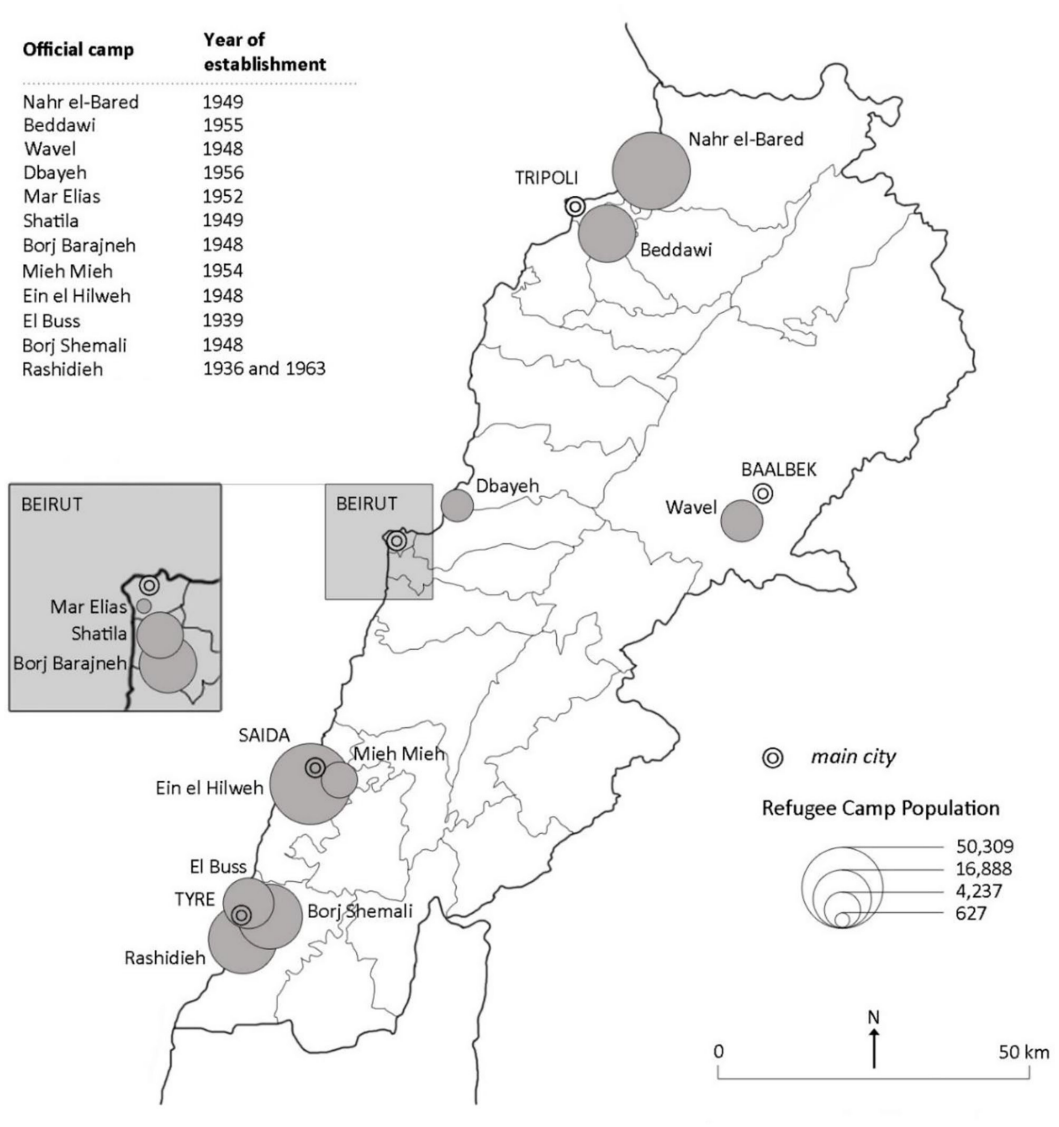
Lebanon's Palestinian refugee camps (Habib, 2012).

When attempting to downscale the aforementioned theoretical discussion on Palestinian refugees in Lebanon, it is important to indicate that the Palestinian refugee case is one of the longest, and therefore most protracted, refugee cases in modern history. Yet this refugee case is unique in terms of its exclusion from the international protection umbrella and host community regulations (Takkenberg, 1998). Lebanon, a small country of just over 10 thousands of square kilometers, is known to have the world's highest refugee per capita ratio (UNHCR, 2020). In addition to a Syrian refugee population estimated at around 1 million, this small country hosts 12 officially registered Palestinian refugee camps (as shown in Figure 1) and 16 unofficial Palestinian gatherings with circumstances quite similar to refugee camps (UNRWA, 2015).

From the beginning of the crisis in 1948, to the arrival of 75,000 Palestinian refugees to Lebanon, and with the purpose of facilitating the provision of humanitarian assistance, Palestinians were forced to move collectively into camps, either by Lebanese authorities or by circumstances dictated by their precarious livelihoods (Peteet, 2005). These refugee camps are either public or private properties rented by UNRWA to host the Palestinian refugee population. Refugee camps have been rented on the basis of a 99 yearlong contract, end of which is approaching and is concerningly anticipated by all stakeholders. UNRWA, referred to in the local Palestinian community as “the witness to the Palestinian crisis,” is a UN agency established solely for the assistance and employment of Palestinian refugees in Lebanon. UNRWA is a unique agency in terms of its commitment to the assistance and employment of only one group of refugees: Palestinians (UNRWA, 2020b). UNRWA provides a space of living for Palestine refugees by renting the area of refugee camps from the Lebanese government and private property owners, in addition to the provision of basic services such as health and education (Hanafi et al., 2014).

Unlike most of the world's refugee camps, and also as an exception to Palestinian refugee camps in the middle east, Palestinian refugee camps in Lebanon are external to the Lebanese government's sovereignty and responsibility. Due to the establishment and then the annulment of the Cairo Agreement—an agreement reached in 1969 between the Lebanese State and the Palestinian Liberation Organization (PLO) to arrange that Lebanese state was governing outside the refugee camps, but the PLO was governing inside the Palestinian refugee camps—Palestinian refugee camps in Lebanon are excluded from the responsibility and control of the Lebanese Government (For further information on this legal lacuna see Hanafi and Long, 2010). Therefore, the organization of the camps in Lebanon today is rather autonomous and is organized mostly by the camp popular committees. Almost in every camp, committees formed of camp dwellers are either appointed or agreed upon by representatives of Palestinian Political parties who are active in that camp. Ideally, the role of these popular committees is to supervise matters of camp security, conflict resolution, utilities and general organization. However, these committees are seen as inefficient, adherent to their own political agendas and rather corrupt, rendering refugee lives in Lebanon even more difficult (For Further information on this see Abou-Zaki, 2013).

Palestine refugees today remain stuck in the same legal and spatial limbo that they were pushed into 72 years ago. Palestine refugees are categorized as “stateless,” meaning that they lack citizenship of a recognized state (see also Shiblak, 2006). And since the Lebanese state has no specific consideration or hospitality regulation for their particular statelessness, Palestinian refugees in Lebanon receive the treatment of foreigners with no recognized state documents, thus depriving them from access to the most basic life elements: labor maker, health and education (UNHCR, 2016). Palestinian refugees in Lebanon today cannot exercise over 30 professions including those in the fields of medicine, law, journalism, technicians, engineering, tourism, transportation and taxi driving, education, accounting and trade in addition to many others (see also International Labor Organization, 2012). As a result of this specific exclusion practiced by the Lebanese government, over 83% of Palestinian refugees in Lebanon do not have a legal form of work, and Lebanon has the highest percentage of abject poverty among all five areas of work on UNRWA in the middle east (UNRWA, 2015). However, because of their prolonged existence and extended suffering, Palestinian refugees in Lebanon have developed their own sense of survival. Lebanon's refugee camps seem to have lost their temporary character and to have become more like permanent solutions (Martin, 2015). Ever since then, Palestinian refugees have never stopped seeking home, in all creative possibilities of the term.

## The Research Question: A Home in Transit?

The issue of homemaking in the Palestinian refugee space is often overlooked and seen to contradict with the temporariness of refugee camps. The exclusion of Palestinians from the socioeconomic life in Lebanon has been a systematic process that aims to prevent the permanent settlement of Palestinians in the country. Therefore, speaking of any permanence or homemaking in Lebanon would invalidate the government's efforts to freeze Palestinians as an isolated and temporary community. By keeping Palestinians excluded from socioeconomic life, they are kept in a state of waiting, they are temporary, and they are not allowed to make a home out of this transit stop. However, the process of homemaking could occur inevitably despite the temporariness. Elisabeth Habib, a Lebanese researcher, stated during her ethnographic field work in Palestinian refugee camps:

“The narratives around refugee camps in Lebanon only relate to the violence and struggles in the camps, to feed the Lebanese resentment against the Palestinians, and it is difficult for anyone who hasn't visited a camp to imagine that people are living there, accomplishing everyday chores, working and studying, making friends, marrying and starting a family.” (Habib, 2012, p. 2)

Authors like Diana Allan, who has performed extensive ethnographic work in Shatila, writes that much of the writing, research, and advocacy work in the camps in Lebanon tends to focus on the violent past and struggles of these communities, while very few have explored how refugees imagine the future and what their hopes and aspirations might be (Allan, 2013). In a way, the image of refugees as agents, capable of planning and acting is highly overlooked for the sake of focusing on refugees as sad victims. Therefore, this paper also serves as a way to verify the way Palestinian refugees make life, build visions and establish home in Lebanon's refugee camps, despite the temporal and spatial suspensions that they are deliberately put in by the state, while maintaining a connection to their Palestinian identity. Allan has also addressed the issue of Palestinian refugee camps in Lebanon in her book “Refugees of the Revolution,” where she illustrates various mechanisms that refugees utilize to cope with chronic uncertainty. Allan's book 2014 was indeed an important resource to refocus the lens on the camp as a political economy through demonstrating the kinship-based survival tactics, historical claustrophobia, and even dreams of refugees in exile (Allan, 2014). The main question of this article thus asks how protracted Palestinian refugees today make home where they are specifically asked not to: in permanently temporary spaces in Lebanon. Unlike the mainstream literature, which highlights mainly the poverty struggles and the humanitarian assistance short-fallings within refugee camps, this paper attempts to explore how refugees survive these short-fallings and cope with it in their own ways, reproducing their home in many different ways. Therefore, this paper will attempt to understand briefly the social reality of Palestinian refugees in Lebanon, but only to demonstrate how Palestinian refugees respond to this social reality through different coping mechanisms and explain the livelihood efforts of camp dwellers who have been in a permanent state of waiting for the past 72 years.

## Methodology

In politically sensitive spaces, ethnographic production is a subjective self-centered process that is highly shaped by political protection and modality of access to the field (Carpi, 2020). Authors such as Allen (2013) have long criticized how investigators visiting Palestinian spaces have been trying to explain the problem from their own perspective, and the author argues for ethnographic recording, like her own, which reports people's lives and beliefs. Having lived in Borj Albarajenah refugee camp most of my life, I have access to the field, interaction with the environment, and thus knowledge production on Palestinian refugee spaces: a process that has been privileged by long term insider view and dynamic membership. Much of this research has been based on participant observations and personal recordings that I have collected during my 26 years of watching and experiencing how the camp evolves. However, additional fieldwork was undertaken in the summer of 2019 during a return trip home, which included ethnographic practices of observation and around 10 semi-structured and unstructured in-depth interviews with Palestinian refugees living in the Borj Albarajenah refugee camp in the Lebanese capital. The recordings included other forms of conversing with the camp as well, through interactions with the camp's geography (neighborhoods, squares, walls), productions (movies and local publications) and even conversations of a personal, intimate, and familial nature. The content of these interviews and observations, to be analyzed in this paper, offer insight to and deduce the dynamic relationship between refugees and their space. The recorded quotes and practices of refugees shall be analyzed in light of my own impressions and understanding as a local dweller, in order to verify the general theory of protracted refugee homemaking in the case of Palestinian refugee camps in Lebanon. Listening to refugee perspectives, in their various forms, is essential for understanding factors that shape refugee self-reliance, as livelihood and well-being are concepts to be perceived in their own context-specific manner (Carpi et al., 2017). The Covid-19 lockdown has hindered further plans of work on the field, but further follow up interviews have been performed remotely through video calls during May 2020. The research also included archival research on the socio-economic and legal status of Palestinian refugee camps in Lebanon, mainly from the publications of UNRWA, in addition to research on refugee cases from international literature. Needless to say, only digital resources were accessible in performing the literature review.

The most important literary source for this paper will be the conceptual framework of homemaking created by Fábos and Brun. The various meanings of home, Home and HOME explained by the authors of “Making Homes in Limbo? A Conceptual Framework” will be utilized to understand the daily practices of Palestinian refugees as processes of homemaking and not only as survival mechanisms. We will see that the simple efforts that Palestinians undertake are more than just day to day efforts but rather are ways they make home of this space where they uncomfortably dwell. The different meanings of daily immaterial and material elements of the camp can be re-explored, reconstructed and revisited in the light of the home-making framework, thus allowing us to find permanency and even safety in one of the world's most unstable spaces: Lebanon's Palestinian refugee camps.

## Exploring Life in the Space of Exception and Protracted Refugee Spaces: The Vertical Survival Mechanism

The history and circumstances of construction of refugee camps vary (Pérez, 2020), and yet all camps in Lebanon today share similar realities in terms of legal constraints, organization and evolution schemes. Despite the many demographic and political changes that could summarize the camps over the years, the current geographic boundaries of these camps remain identical to their establishment (UNRWA, 2011). Refugee camps were immediately built after the exodus of Palestinian refugees as temporary spaces, but tents slowly evolved into concrete structures over the years (Chaaban et al., 2010). According to Mauriat, in most cases Palestine refugees built their new houses or more permanent structures on the exact spot where their arrival tents stood (Mauriat, 2001). Elisabeth Habib reports, through her interviews with camp elders and while recording their stories, that it was only 6 years after arriving to Lebanon that the Palestine refugees lost hope of an imminent return to their land (Habib, 2012). Habib further describes how living conditions under the weak and degrading fabric of tents, became extremely difficult: “The tents, aimed at offering a provisional shelter were neither adapted to the winter, nor to the summer climates. Not trying anymore to survive while waiting an imminent return, the refugees wished now to settle in decent conditions, to build themselves a community” (Habib, 2012, p. 25).

As a result, and despite all constraints, the Palestinian refugee community in Lebanon has grown from a population of 75,000 refugees who arrived in 1948 to around half a million Palestinian refugees residing in Lebanon today (UNRWA, 2020a). Failing to solve the refugee crisis, the camps never stopped growing and changing. Walking around the camps today, I can understand much about the camp dwellers through the camp geography. I first notice the naming of camp neighborhoods after the names of Palestinian villages and cities. For example, families from the village of “Tarshiha” (which is a Palestinian Village heavily bombed and looted during the 1948 conflict) have mostly grouped in a neighborhood and named it “Jouret El Tarashha” جورة التراشحة which means the “pit of people” from Tarshiha. Other areas of the camps are named after the big families residing there such as “sahet el ashwah” ساحة الأشوح which means the square of Al-Ashwah family, a big family in the refugee camp, and alludes to the priority of strong family community and maintaining connections. Important camp facilities are also named after places in the occupied Palestinian territories, such as “Haifa Hospital” in the Borj Albarajenah camp, and “Acre Hospital” in the Shatila camp, both of which are named after occupied cities on the Palestinian coast. When I walk through the alleys of the camp, its narrow walls push me to read every single poster. The walls are filled with pictures of Palestinian martyrs and the skies are covered with pictures of Palestinian political figures. Looking around, one can also notice posters celebrating or mourning the different Palestinian occasions, such as “land day” and Yasser Arafat's memorial. I come across a person on the side of the alley handing out flyers and invitations to a Palestinian folk music event hosted by a local arts organization (Al-Jana), and another who hands out stickers, produced by Hamas local office, condemning the violations of Al-Aqsa Mosque. I see announcements for activities and events, on and off the topic of Israeli occupation and Palestinian resistance, and the posters declaring solidarity of the camp dwellers with the people of Gaza are a common across the camps of Lebanon. People of similar roots in Palestine have grouped together and established their space collectively by building common homes near each other, but they have also created a home denoted by their homeland that reflects their collective history, family story and personal identity. Camp dwellers therefore reproduce their backgrounds and even their own narratives in their new homes, thus creating their own space as they desire. Abdulrahman, a middle-age camp dweller, says to me in an interview:

“We know very well who we are. Why would you use the term stateless? We are Palestinian. In my opinion, if you want to see the real Palestine, you come to the refugee camp, not even to the Palestinian territories, they have been influenced by occupation and politics, but here, here you find the isolated group, thus the preserved identity, the Palestinian identity.”

Looking upwards, I can clearly see how Palestinian refugees in Lebanon have worked toward improving their living conditions in this temporary space, and understand how camps managed to survive and grow demographically within the same number of square meters. Legally, refugee camps have found no horizontal space to expand, therefore resorted to expanding vertically, thus creating many additional layers to the original primitive structures built in the 50 s. Taking the Borj Albarajenah camp in Southern Beirut as an example, the name of the camp denotes its stunning vertical growth: Borj Albarajenah برج البراجنة means “Tower of the towers.” Due to the lack of planning, organization and development policies targeting Palestinian refugees, refugees took it upon themselves to improve their lives by establishing extra space to live in and shelter their growing families.

These structures, which evolved from tents to concrete and then primitive metal buildings from the first 40 years of the exile, have now been regarded as opportunity to build on, taking the open space of the structure roof as a space to move upwards, therefore establishing second, third and in some cases even fourth additional floors. Families often build additional floors in order to give the boys of the family an opportunity for marriage and establishing a family of their own in the newly built layer. However, recently, camp dwellers have been using this mechanism also to create a source of income. For example, today house owners either sell their own empty unconstructed house roof, for a client who wants to build a house on it. A wealthier camp family would build the roof as an apartment then sells or rents it to another client for a higher amount of money. In other cases, the family would build the extra floor to inhabit a sunnier location with better air circulation, while renting the lower floor into a shop or workspace. In this way, the family would have a better living environment, obtain a source of rent income, and provide the camp dwellers with space to perform work activities. Alya (dweller of the Borj Albarajenah camp) says that she preferred building an additional floor and moving up into a higher level because it was a good protection mechanism from the winter flooding which drowned her ground house and destroyed her furniture every year. In addition, she says that a higher floor makes her and her family safer from the roaches, mice, rats and cats which easily enter houses on the ground floors but now cannot enter her upper house as easily. She blames the primitive sewage systems for the spread of rodents and other sanitary problems, but she explains that they have found this upward rise of buildings to be an effective coping mechanism.

In an interview I have conducted with Hussein- a dweller of Borj Alabarajenah camp in August of 2019, he says:

“My age? Oh, I do not know. I think I was born in 1950. My family had just been expelled from Kabri (village in Palestine) and living in tents in Beirut, they could not care less about registering my birth. But I know that I was born in winter, because they said that the thunderstorms were so heavy that night, that the tent flew off my mother while she was giving birth to me. It was not going to be a problem until all the other tents of the camp flew away and suddenly everyone was watching what's happening between my mother's legs. The other women of the camp ran with bed sheets and blankets towards her, formed a circle and covered her birth process until I arrived safely to the world. And since then, this camp has been all I have ever known. I worked some handy crafts in my youth to make a living to me and my wife, I built a second floor over my parents' house to my own family, but I was not lucky enough to have children, so I began to think of a profession that will guarantee me some financial security with older age. So, 30 years ago, I started my tobacco shop here in the Ashwah square in the camp. I rented a ground floor of this house for 100 thousand liras a month, and the original dwellers just moved upstairs. The smugglers bring us cheap tobacco from Syria, and we sell it here to camp dwellers for competitive prices. Sometimes I even get clients from outside the camp who come for cheap tax-free tobacco.”

It is worth mentioning that all structures in the camp areas are not legally owned by camp dwellers themselves, as the camp is only rented by the UNRWA from the public/private landowners. However, an entire market of property buying, renting, and selling has been established on this space by camp dwellers who are not actual owners of the properties involved in the exchange. Nevertheless, for organizational purposes, sale and purchase activities are recorded at the camp popular committee in a way to protect dwellers from potential property conflicts.

The camp roofs are not merely an economic commodity by which camp dwellers obtain income, but rather also a way to secure more space for social bonding. Many camp dwellers perform the required maintenance on their roof and place high borders (balusters(in order to use the area as a collective social space, especially for summer nights. In Palestinian refugee camps in Lebanon, social ties are evidently strong for the observer, and most social events (weddings, funerals, special events and family gatherings) are normally crowded, and thus more conveniently hosted on the roofs. For many women, it has become the place where they share the morning coffee gathering ritual with other women known as *Sobheyye* صبحية,, which means “practicing a morning thing,” and it refers to the act of women gathering for coffee, chats and cup readings every morning. For others, it has become the regular location for the daily *Sahra* سهرة, which means “being awake for spending time for an activity or gathering in the evening,” because it is the only place to avoid overheating of damp and crowded camp buildings. Ibtihaj, who is a grandmother of 6 children, says to me:

“*In exile, you have no land, no assets and no state protection, your family and community are the only sense of protection that you have. A normal citizen of any country goes to the hospital when he gets sick, we cannot afford to do that, we go to the neighbor who knows how to make herbal remedies. Most people, go to the movies or even on a holiday when they get bored, we cannot do that, we just go to each other's houses or roofs.”*

Such practices of gathering in such spaces is not only an evident of space agency but also of the mechanism by which the social fabric of Palestinian refugees is formed. Through the creation of the camp spaces as a home or even as just spaces of utility, bonding occurs among refugees and grows as a way to cope with lack of space and activities.

A large percentage of camp dwellers go further than just merely considering this as a “breath out” space but rather take the effort to transform into recreational spaces. Because no parks or gardens exist within the camp area due to the lack of space, many camp dwellers have decided to transform their roofs into small-scale gardens, where they place many potted plants and sometimes small animals such as birds, bunnies or turtles in that space. It is common also among dwellers to set up vertical structures around the roof as a pergola, where grape leaves would form a coverage net, providing a soothing atmosphere similar to a garden. It is because these human needs for leisurely spaces, or open spaces are not normally available within the camp ground, that refugees are forced to seek them in alternatives areas of the camp, or rather establish them on their own through unconventional methods and locations.

A major problem with this expansion is that the proper construction base was never built to be expandable. The original building structures of these camps on the ground floor were initially built for temporary short term settling with no need for long-term endurance, or for additional concrete layers. The construction material and the construction plans were primitive, unprofessional, mostly done by locals who have no engineering skills or access to quality construction material, and thus this situation produced temporary homes that could accommodate dwellers for only a few more years until the eventual resolution of the refugee crisis. However, because the refugee crisis was never resolved, additional construction was erected, and no possible considerations could have been made to the durability of the building base to handle 3 or 4 additional floors. Therefore, the entire building is a true safety hazard to its dwellers and its surroundings.

To make matters worse, the Lebanese legislations had prevented the entry of any construction material to any Palestinian refugee camp in Lebanon, strictly until 2007, in an attempt to control the expansion of the camp, not taking into consideration the demographic growth of the refugee population which could be left unsheltered (Amnesty International, 2007). Even though the law had been eased after 2007, allowing Palestinian refugees to obtain construction permits to bring in construction material, it remains highly difficult for Palestinian refugees to obtain the required construction permits and because it is almost impossible to meet the ministry's construction standards in such exceptional dwelling space. However, smugglers of construction material and cement continues to provide the camp with needed material through unnoticed entry spots during hours of less surveillance. Technically, most camp structures are illegal but exist today due to a network of connections based on bribery (Halabi, 2004).

Strict control of entering construction material to the camp is not the only problem of the camp's vertical expansion. The camp expansion is creating higher constructions in terms of altitude, covering the small camp atmosphere and sky/sun visibility, thus inhibiting air flow, causing higher dampness and darkness on the ground level of the camp. Therefore, residential lower floors and camp pathways are suffering from extreme darkness, dampness and mold, and low air circulation. According to UNRWA, 78% of Palestinian households in Lebanon's refugee camps are affected by dampness, 62% suffering from water leakages, 52% suffering from poor ventilation, and 55% are affected by darkness (UNRWA, 2015). The construction sometimes also requires building additional concrete enforcements to the lower constructions in order to hold the additional floors, thus making the pathways and spaces on the ground smaller and narrower, further constricting the already-tight walking paths and limiting further mobility space for camp dwellers to roam through their camp. Due to the additional, unplanned, and unorganized construction, pathways have become so narrow, and the ground levels become uneven and irregular. Nadya Fakhereddine writes her story in Arabic as a Palestinian refugee in the Book “Eleven Stories from the Exile” saying:

“Since I became a mother, I began to hate the winter, and the camp. It became especially hard for me to walk around the camp once I began to need to push my daughter on a stroller on the uneven ground and through the narrow pathways. Instead I am obliged to -unsafely- carry my daughter everywhere, which is especially difficult in winter, when the ground is flooding in sewage water, and the sky is pouring heavy rain over so many electric cables in sight.” (Yāsin et al., 2017, p. 78)

Due to the narrowing of the pathways and the lack of actual access roads that reach the internal parts of the camps, no cars, buses, or trucks can enter most Palestinian refugee camps in Lebanon. The refugees resort to small motorcycles as the most efficient mode of transportation within the camp to transport adults, children, goods, and even furniture. This mode of transportation is also another way that Palestinian refugees take charge of their own space and mobility restrictions. Director Alaa El Ali even created a short film in 2014 titled “Journey of a Sofa,” which tells the story of young Palestinian refugees who underwent a long and troublesome journey to navigate a sofa through the difficult pathways and structures of the Shatila camp in Lebanon (El Ali, 2014). The short film presents not only the struggles of Palestinians in leading a basic and normal life, but also their persistent attempts to make a home despite the difficulties.

Another serious problem of this vertical unplanned growth is the unorganized sewage and electrical connections that must accompany the erection of new floors without authorization or control. Lacking authority control or planning of the additional sewage connection or electrical cables, the new cables and tubes make their way in spontaneous manners along the narrow pathways, through the roofs, and in between houses with little consideration to safety. In fact, in the Borj Albarajenah camp alone, 48 people have died in the past 5 years due to electrocution in the pathways. The combination of water flow with high voltage wires often leads to a true hazard, especially in winter times due to heavy rain and thunderstorms.

Vertical expansion within the borders of Palestinian refugee camps is not only a matter of staying alive, but is also a matter of the afterlife. Due to the limited area of the camps, the spaces to bury the dead within the camp are similarly fully occupied. According to the Lebanese religious authority (Dar Al Iftaa), deaths within Palestinian refugee camps must be either buried within the camp area or be taken away to a remote burial space in the southern mountain areas (to which Palestinian refugees have limited mobility). As a result, even the burial spaces (tombs) became vertical. Camp dwellers would prefer to maintain rituals of visiting and bonding with their deceased, so they have resorted to a religious Islamic fatwa (a ruling on a point of Islamic law) which legalizes the burial of one body on top of another body within the same grave. This means that bodies can now be buried on top of each other in the same tomb as long as a certain blood relation exists between the deceased individuals. With time, the act of burying individuals above each other in the same grave has become a bonding ritual, a sentimental practice that allows camp dwellers to strengthen their ties with their lost loved ones. For example, people today think thoroughly about where they want to be buried when they die; they plan and request specifically ahead of time: “With who do I want to be buried, on top of whose body do I want to spend eternity?” Thus, it is not rare to encounter elders who wish to be buried on top of their mother to demonstrate deep love toward a person they valued in their life. In my interview with Nahida, of the Borj Albarajenah camp, she explains that she “cannot think of a safer place to rest forever better than on the body of her beloved mother.” Burial in the camp cemetery has also become a symbol of resistance. Samer, from the Shatila camp, refers to many Palestinian fighters and revolutionary icons who have been buried in the camp cemeteries and explains the symbolic value of the camp to Palestinians and supporters of the case worldwide. He specifically mentions Franco Fontana, the Italian fighter who volunteered with the Palestinian revolution in the 80s and requested to be buried in Palestine, or in Palestinian refugee camps. Fontana lived his final days with Samer, and was buried in the cemetery of Ein El-Helwe camp in the south of Lebanon in 2015. As such, camp dwellers have come to transform these coping mechanisms into an opportunity for bonding, love and social cohesion that transcends even into the afterlife, but it is also a form of resistance and sense of belonging to their Palestinian identity.

## A Temporary Home for 70+ Years: Discussion With the Feminist Framework of Homemaking

Palestinian refugees are stuck between homes. Neither are they capable of returning to their occupied territories, nor are they able to officially settle in their host community due to the legal specificity of the Lebanese constitution. From the Lebanese point of view, the naturalization of the Palestinians and their assimilation to the Lebanese society would, first, recognize the legitimacy of the State of Israel and, second, shake up the demographic balance of the country, both of which are actions that the Lebanese state do not anticipate making in the near future, according to a 2012 report issued by the Ministry of Environment: “It is important to note that both the Palestinians and the Lebanese reject complete assimilation of refugees into the Lebanese State. This is mainly because assimilation conflicts with the Palestinians' UN sanctioned right of return.” (Ministry of Environment, 2012, p. 19). As a result, Palestinian refugees remain temporary, with no option of permanently settling in Lebanon and calling it there home through a legal citizenship or residency. However, Palestinian refugees carry out practices of home, Home and HOME making in refugee camps, thus reproducing a sense of belonging to the waiting-zone despite the socioeconomic challenges and host-community restrictions.

As Brun and Fábos noted in their work on protracted refugees, homemaking as a process takes place, despite the rigidity of circumstances. People make a home when they attempt to recreate something from their nostalgia, improve their material conditions, and plan a better tomorrow, regardless of the deep uncertainty concerning that future (Brun and Fábos, 2015).

We can deduce from the insights to the Palestinian refugee life that Palestinian refugees have established “home-Home and HOME,” all three elements of homemaking created by Brun and Fábos, in their waiting zones despite the deliberate legislative system aiming to freeze them. They have created the first pillar of the framework as “home” by building houses in which they can feel safe and comfortable, rather than settling for short-term housing solutions that they had previously. They created “home” by planning and securing sources of income that could guarantee them a dignified life along the long road ahead, by creating additional spaces to survive and enjoy, by making families and expanding the span of their human communication. They constantly attempt to improve their economic condition through finding new resources of income such as building extra stores and levels, and indeed plan for the long-term futures in the camps, such as situations of old age and family housing despite the uncertainty about their future. Through the formation of a micro-economy, refugees have been exploring, producing, and exchanging space in a situation where space is both a rare and valuable element. The creation of this non-conventional market, which is unique to the camp space, reflects the need and desire of refugees to produce better daily living conditions but also long-term financial security. The refugee's efforts to building an economic activity to produce an income, and the investment of this income in improving the space and living conditions, is a process that reflects home-making. These two elements imply a how refugees view this space as a “home”: the space where dwellers attempt to improve their material living conditions. Stories such as that of Hussein Balkis's shop demonstrate that these refugees make a “home,” as the daily practice of improving the quality of life, through attempts to prepare for a long future in refugee camps despite knowing that they can be permanent there. In other words, they maintain the idea of home in Palestine through a Palestinian identity, yet they maintain the commitment to making a home in this refugee camp. Combining both creates a unique notion of home in many ways. We can view how Palestinian refugees turned this space into a “home” through their efforts of denoting and organizing it, through establishing relationships with the Lebanese and even Syrian surrounding environments and through exchanging and smuggling material. Expanding and exchanging are both a method of survival and homemaking. By establishing a mechanism to make life better for the long term, refugees have been asserting their connection to the space despite being constantly pushed to abandon it, a space which they are repeatedly told as temporary. A camp space becomes meaningful when camp dwellers interact with the space, invest their emotional and material assets (Capo, 2015). In these particular camp spaces in Lebanon, camp spaces become meaningful when refugees create memories there, give them meaningful names, and establish a personal relationship with them. By investing their time and efforts in changing the place, they implement their vision of home in that space.

Palestinian refugees also established “Home” in the way they maintain their identity as Palestinian and hold true to their traditions. Examining the camp space, we could notice how Palestinian refugees grouped each other according to their lives back in Palestine: families from same villages chose to dwell in neighborhoods named after them. They recreated the mapping of their original villages and cities in their new host community as an attempt to reflect their original home in their new one. This can be seen further than just a coping mechanism but also as a tool of identity preservation and national commitment to the collective Palestinian homeland. This nostalgia and connection to the homeland brings us to recognizing Brun and Fábos's concept of Home, which is summarized by the sentimental lingering to homeland, and commitment to its identity. This enables us to view refugee camps as “Home,” because refugees recreate their camp space with the idea of their Palestinian homeland as the inspiration of the Homemaking process. The Ein Elhelwe refugee camp in the south of Lebanon is even dubbed as the “capital of the diaspora,” which indicates that Palestinian refugees view their diaspora as a “country” with a capital, with cities and citizens, like a temporary parallel to the state of Palestine. This condition of being in between homes can be confusing for the external viewer, but these people have established life in these camps on the Lebanese territories while maintaining an identity of a land to where they wish to return. Refugees in the camp still value social cohesion, daily communication, and family relationships because it is the only connection they have to their Palestinian roots. Today in the camp, I see family members living in the same building with their parents not only for lack of space, but also for the significance of connection to elders and youth of the family. Similarly, connection to elders was evident in the organization of graves where individuals were willing to share the small space for eternity. Refugees share space, meals, and activities, and often perform those activities based on their Palestinian traditions. More evidently, I was able to view the Home-making process through observing the 3rd generation of Palestinian refugees, who were born in and lived their entire lives in Lebanon refugee camps; they, therefore, had no means of knowing their original home. How do 3rd generation refugees reproduce their living space and Homemaking according to a homeland that they have never seen? The answer is that the strong commitment we see among 3rd generation Palestinian refugees to their Palestinian identity is proof to the strong bond between successive generations within the camp. One can understand from the strong relations that Palestinians have with their families and elders is that this camp is “built on stories,” where the process of making this camp a “Home” is highly correlated to the ideas and identity passed on from the camp elders to the successive generations through family gatherings and children upbringing. By gathering, sharing stories and heritage, establishing a common identity, they make the camp a Home. Therefore, the process of Homemaking is rather produced by the idea of a former home (Palestine) that becomes the new space of exile, where refugees are stuck, ultimately transforming the camp into an attempted reproduction of their homeland in Palestine.

Palestinian refugees in Lebanon also establish HOME in its political sense: they speak of “Palestine” without using other vocabulary to signify its current political status. They reassert their right to return, to organize events and activities to reassure their commitment. They reject the recognition of the Israeli state, and they view their existence in these camps as a form of resistance. Refugees in the camp speak in pure Palestinian dialect and follow the Palestinian political news more than they follow the Lebanese local news, knowing that the changes in Palestine influence them directly, despite being physically external to the geographic Palestinian zone. This allows us to see the notion of HOME, where the connection with the homeland is rather not only sentimental but also political. Even though they live in Lebanon, they are technically geographically external to events happening on Palestinian grounds, but still they remain strongly and solely connected to their Palestinian political reality. Furthermore, they are aware that the existence of the refugee camps is itself a hurdle to the occupation of Palestinian lands, for occupation cannot be complete when 5 million refugees are stuck in and outside camps worldwide, demanding to return to their Palestinian lands and refusing resettlement in new countries. By remaining in these refugee camps, the Palestinian-Israeli conflict cannot be solved. This is because any suggested solution for the conflict must include some resolution for the 5 million Palestinian refugees scattered around the middle east (Masalha, 2003). If a two-state solution is established, the refugees remain unsettled, and thus the solution remains incomplete, leaving space for further tension. Therefore, these camps are intrinsically hubs for political discussion and are contributing to the political negotiation-even if only passively- by merely existing. Thus, the refugee camps have a political meaning to the dwellers, making them more adherent to this space where their struggle becomes of more meaning and value, thus is becomes more of a HOME. Palestinians, like most displaced peoples, are thus inevitably political communities by nature and thus have political claims which are both international (directed at the global apartheid structure) and national (identifying state responsibilities toward them) (Feldman, 2018). By being in refugee camps, the Palestinians are including themselves in the Palestinian space despite being external to the geographic space of Palestinian territories. They are maintaining themselves relevant to the Palestinian-Israeli conflict and remaining an integral part of the negotiation merely by surviving and HOME-making in the refugee camp.

Observing the daily life and long-term practices of people over 72 years, we could thus verify how the camp structures transform from (almost) houses to full homes, and the camp itself becomes a collective home, gathering those who belong to the same community and collective cause. Through solidarity, social bonding and sharing of a limited space and resources, refugees are reproducing the community from which they are displaced, creating a miniature model of the country they left behind, as this is the strongest element uniting them. This isolated community establishes stronger bonds and firmer social ties, based on a common belonging to the occupied territories of historical Palestine and creating a chance for solidarity and cooperation to relieve the community from difficult socioeconomic circumstances. We can therefore see how the common struggle for returning home, or having a home, unites the camp dwellers and brings them together to establish coping and protective mechanisms that could ensure survival through the period of protraction. The development of these mechanisms is a proliferation of the way spaces of transit and waiting become organized in their own ways, through their own modes of agency, just like lasting cities, even in the absence of formal planning (Agier, 2011). As we could observe from the activities within the community, this camp has not only created its own networks and organizational channels, but the camp has even created its own microeconomy through the trade of spaces and building facilities. The agency of refugees over their space rendered it into an unconventional urban phenomenon in which the dwellers make a living and conduct all practices of homemaking.

## Conclusion: “I'm Home Until I Get to Go Home”

Refugee camps are normally established as safe spaces where groups fleeing danger seek refuge and protection. However, the view of life in Lebanon's refugee camps invites us to reexamine the meaning of a refugee camps as a protective mechanism. Being called by the media as “Lebanon's most dangerous refugee camps” (Dowling, 2019), it is difficult to imagine humans making home of such spaces. However, the unusual organization of these spaces and daily practices of refugees within these camps provoke us to reconsider the notion of refugee camps as “homes” in the light of their ongoing protraction and resilience.

Despite the difficult circumstances and tough restrictions imposed by the Lebanese government, we can view that refugees unreluctantly conduct the practices of homemaking, even in their own ways and with minimal resources, which could challenge the safe or conventional methods of livelihood. They establish their homes through expanding vertically, thus making home not only for themselves but also for the upcoming generation, to find home and survival methods in this camp. Thus, they are challenging the temporariness of these camps by paving the way for some form of long-term settlement, despite the perpetual desire to return to their original homes. Another way of making “home” is through making this camp space a mechanism of income generation. Furthermore, refugees in Lebanon's refugee camps make “Home” by reproducing their Palestinian homeland in their exile through traditions, geography, and language. And eventually, by being a symbol of resistance to the political plan of dismantling the Palestinian state, the Palestinian refugees are making “HOME,” a place by which they pose their own political contribution to the struggle which concerns their future.

For protracted refugees as the Palestinians, they are supplied with short-term solutions and temporary shelters which transform from aid solutions to actual problems with the passage of time as the crisis exceeds being temporary. Palestinian refugees are given ephemeral solutions and equipped with survival mechanisms which are not designed to last, from both the humanitarian government and the host community legislations. Instead they are promised that their refuge will not last, and that these policies and mechanisms will not be needed for long. Therefore, they suffer the most when these short-lived mechanisms expire, causing true humanitarian suffering. When their refuge lasts longer than expected, with no proper structures in place to address the humanitarian crisis, the gap produces a space for unusual coping mechanisms which are admirable in terms of innovation yet are very challenging and must be examined in terms of sustainability.

The Palestinian refugee crisis is one of the longest in history, and therefore must be addressed through the implementation of the UN resolution number 194 article 11 addressing the return of refugees. However, the current situation highlights the role of the host community and the humanitarian government in limiting, overlooking, and restricting the development of the refugee camps. The local government's policy of containing camp growth is not effective, but rather counterproductive, as one can observe through this paper how refugee camps grow and expand into city-like structures, where dwellers are making home through novel coping and homemaking mechanisms. This scenario of coping is not to be romanticized. The fact that the camps and services have not been expanded to cope with population growth has resulted in severe overcrowding, which negatively impacts refugees' quality of life and health, as well as the general environmental and sanitary conditions of the camps in Lebanon. Decaying infrastructure, lack of recreational spaces, insufficient access roads, very limited natural light and ventilation, in addition to shelters that were designed to be temporary but are still standing and upwardly expanding, all pose serious safety and health risks (UNRWA, 2011). The high cost of materials, combined with the restrictions imposed on bringing construction materials into the camps, means that refugee families have been unable to carry out substantial repairs or maintenance (UNRWA, 2011), thus putting camp dwellers in danger without keeping them from natural community expansion and refugee homemaking.

The focus of this paper, on being temporary for 72 years yet still being able to make home and maintain a sense of belonging to a remote homeland, gives a 2-fold challenge to public policies concerning refugees: a challenge for host community policies and a challenge to international policies toward refugees. In the case of Palestinian refugees, Lebanon's policy of keeping refugees from settling in Lebanon has failed as Palestinians have established homes in these refugee camps, and Israel's policy of preventing refugees from return has failed as refugees have maintained their Palestinian identity and sense of belonging. Therefore, homemaking is a process that occurs inevitably through the human connection to both space and community, despite public policy arrangements. Furthermore, the study indicates the importance of both the access of Palestinians in Lebanon to basic rights and public services, and the implementation of the Palestinian right of return to their homes, knowing that one does not contradict the other.

In the current year, and more recently within the Lebanese recent revolution of October 17th (which started in 2019), solidarity has risen with Palestinian refugees in Lebanon, and especially on social media, claiming the rights for dignified life until the Palestinian refugee problem must be resolved in a manner of justice. Activists have been repeating the slogan “A Palestinian is home until he gets to go home,” thus confirming both the rights to enjoy full rights while in transit, in addition to the right of returning home. As Nur Masalha explains, Palestinian refugees themselves insist on ending their crisis into return to their lands. They have long waited repatriation and refused resettlement since their exodus in 1948:

“Among the Palestinians, the belief in return is strongly held. Refugee feelings concerning the ‘dream of return’ are intense. The yearning for Palestine permeates the whole refugee community, and is strongly felt by younger refugees, for whom home exists only in the imagination.” (Masalha, 2003, p. 70)

However, until they do return, refugees demand and deserve a safe and dignified life, with healthy living conditions in the temporary spaces which they have established strong emotions, shared efforts, and made home.

## Data Availability Statement

The raw data supporting the conclusions of this article will be made available by the authors, without undue reservation.

## Ethics Statement

Ethical review and approval was not required for the study on human participants in accordance with the local legislation and institutional requirements. The patients/participants provided their written informed consent to participate in this study. Written informed consent was obtained from the individual(s) for the publication of any potentially identifiable images or data included in this article.

## Author Contributions

The author confirms being the sole contributor of this work and has approved it for publication.

## Conflict of Interest

The author declares that the research was conducted in the absence of any commercial or financial relationships that could be construed as a potential conflict of interest.
